# Long Non-Coding RNA Profile Study Identifies an Immune-Related lncRNA Prognostic Signature for Kidney Renal Clear Cell Carcinoma

**DOI:** 10.3389/fonc.2020.01430

**Published:** 2020-08-20

**Authors:** Zhuolun Sun, Changying Jing, Chutian Xiao, Tengcheng Li

**Affiliations:** ^1^Department of Urology, Third Affiliated Hospital of Sun Yat-sen University, Guangzhou, China; ^2^Eye Institute of Shandong University of Traditional Chinese Medicine, Jinan, China

**Keywords:** immune, kidney renal clear cell carcinoma, long non-coding RNA, overall survival, prognostic signature

## Abstract

Kidney renal clear cell carcinoma (KIRC) is the predominant pathological subtype of renal cell carcinoma (RCC) in adults. Long non-coding RNAs (lncRNAs) are an important class of gene expression regulators and serve fundamental roles in immune regulation. The intent of this study is to develop a novel immune-related lncRNA signature to accurately predict the prognosis for KIRC patients. Here, we performed genome-wide comparative analysis of lncRNA expression profiles in 537 KIRC patients from The Cancer Genome Atlas (TCGA) database. Cox regression model–identified immune-related lncRNAs were extracted for constructing a novel five immune-related lncRNA signature (AC008105.3, LINC02084, AC243960.1, AC093278.2, and AC108449.2) with the ability to predict the prognosis of KIRC patients. Univariate and multivariate Cox regression analyses demonstrated that the signature could act as an independent prognostic predictor for overall survival (OS). With the further investigation on different clinicopathological parameters, we found that the signature could divide KIRC samples into high-risk groups with shorter OS and low-risk groups with longer OS in different subgroups. Principal component analysis suggested that the five immune-related lncRNA signature drew a clear distinction between high- and low-risk groups based on the immune-related lncRNAs. The different immune status between the two groups was observed in gene set enrichment analysis and the ESTIMATE algorithm. Except for AC093278.2, the expressions of the other four lncRNAs expression were significantly upregulated in tumor tissues. In summary, the identified immune-lncRNA signature had important clinical implications in prognosis prediction and could be exploited as underlying immune therapeutic targets for KIRC patients.

## Introduction

Renal cell carcinoma (RCC), accounting for ~2% of adult malignancies, is the third most common malignant tumor of the urinary system worldwide following prostate and bladder cancer (1). Kidney renal clear cell carcinoma (KIRC) is the predominant pathological subtype and represents ~90% of the total cases of RCC in adults (2). Since the clinical symptoms and signs of early stage RCC are often insidious and non-specific, a great proportion of patients are not diagnosed until advanced tumor stages (3). Furthermore, KIRC is known for being insensitive to chemotherapy and radiotherapy and characterized by higher rates of recurrence and metastasis compared to other subtypes of RCC (1, 4). The 5-year overall survival (OS) rate for patients with early stage RCC is up to 90% although OS of those with locally advanced RCC and metastatic RCC could drop to 60 and 10%, respectively (5). Immunotherapy has emerged as one of the most promising modalities against cancer, and recent clinical advances have confirmed its value in urological cancer (6). Thus, investigation on immune-related factors is urgently required.

Long non-coding RNAs (lncRNAs) are a class of transcribed non-coding RNAs (ncRNAs) that are longer than 200 nucleotides in length and do not encode any proteins, which are widely distributed in the cytoplasm and nucleus (7, 8). It is well-documented that lncRNAs are implicated in multiple biological functions, such as cell differentiation (9), apoptosis (10), tumor microenvironment (TME) (11), and epigenetic regulation (12). Recent research indicates that lncRNAs exert a complex and comprehensive regulatory role in cancer development and progression (13, 14). Moreover, lncRNAs have emerged as important regulators of gene expression in the immune system, including but not limited to immune activation and immune cell infiltration (15, 16). For example, lncRNA SNHG1 plays a critical role in the immune escape by inhibiting the differentiation of Treg cells in breast cancer (17). Analogously, NKILA, an NF-κB-interacting lncRNA, promotes tumor immune evasion by regulating activation-induced cell death of various T cell subset infiltrating tumors (18). Other research reveals that oncogenic lncRNA LINK-A inactivates tumor suppressor pathways and downregulates antigen presentation through inactivation of PKA pathways (19). The immune system affects oncogenesis greatly, and immunotherapy has emerged as a promising strategy in cancer treatment (20). Therefore, it is crucial to explore immune-related lncRNAs to predict prognosis of KIRC patients and further guide the proper individual treatment strategies.

In this study, we performed a comprehensive comparative genomics analysis of lncRNA expression profiles in 537 KIRC patients from The Cancer Genome Atlas (TCGA) database. The Cox regression model identified five lncRNAs that are related to immune response. We then constructed a novel immune-related lncRNA signature with the ability to predict the prognosis of KIRC patients, which might serve as potential prognostic indicators and could be exploited as underlying immune therapeutic targets for KIRC patients.

## Materials and Methods

### Acquisition of KIRC Expression Data

Both the entire RNA-sequencing profile data and corresponding clinical information of patients with KIRC were downloaded from the TCGA (https://cancergenome.nih.gov/) database. We downloaded the raw reads and fragments per kilobase of transcript per million (FPKM) data for our study. According to the gene annotations in the GENCODE project (https://www.gencodegenes.org/) (21), the lncRNAs and protein-coding genes were further classified. Subsequently, the detailed clinical information of tumor patients, including age, gender, tumor grade, TNM stage, AJCC stage, and survival status were obtained for further analysis. Similarly, the mutation data of patients with KIRC were downloaded as a mutation annotation format (MAF) file from TCGA database. Analysis, visualization, and summarization of MAF files using R package “maftools” (https://github.com/PoisonAlien/maftools) (22). Considering that some patients may die from non-neoplastic factors, samples with overall survival (OS) data less than 30 days were excluded. In addition, a proportion of KIRC subjects with incomplete data were also rejected. No specific ethical approval and informed consent were considered necessary for all of these data were publicly available.

### Identification of Immune-Related lncRNAs

List of the immunomodulatory genes was downloaded from the Molecular Signatures Database v7.1 (MSigDB, https://www.gsea-msigdb.org/gsea/index.jsp, IMMUNE_RESPONSE, M19817 and IMMUNE_SYSTEM_PROCESS, M13664) (23). To identify the potential lncRNA related with immune-modulating genes, we performed Pearson correlation analysis in the statistical software R (version 3.6.2). The correlation coefficient (|R|) greater than 0.8 was considered as a strong correlation, and *P* < 0.05 was statistically significant. Based on the above thresholds, candidate immune-related lncRNAs were identified and used for further analysis.

### Construction of the Prognostic Signature and Calculation of the Risk Score

To confirm the potential prognostic-related lncRNAs, univariate Cox regression analysis was performed to analyze the association between immune-related lncRNA expression and survival data. Those immune-related lncRNAs significantly related to survival (*P* < 0.001) were selected as prognosis-related lncRNAs of KIRC patients. An HR value greater than one suggested an increased risk; otherwise, it suggested a protective risk. Multivariate Cox regression analysis was employed to confirm target immune-related lncRNAs and its estimated regression coefficients (β) with the lowest Akaike information criterion (AIC) values. We then constructed the optimal lncRNA prognostic signature and calculated the risk score of each PIRC patient on the basis of the risk coefficients as well as the expression levels of target lncRNAs. The risk score was calculated as risk score = β_lncRNA1_ × Expression_lncRNA1_ +β_lncRNA2_ × Expression_lncRNA2_ +…+β_lncRNA1n_ × Expression_lncRNAn_.

### Prediction Analysis of Risk Score Model

All KIRC patients were sorted into high- and low-risk groups with the median risk score as the threshold. We depicted the survival curve between two groups using the Kaplan-Meier method with a two-sided *log-rank test*. In addition, the receiver operating characteristic (ROC) curve and area under the ROC curve (AUC value) were utilized to evaluate diagnostic efficacies. Univariate Cox regression analysis was used to evaluate clinicopathological variables that affect the survival of KIRC patients, including age, gender, grade, AJCC stage, T stage, and M stage. N stage was not analyzed due to lacking a large amount of data. Furthermore, the risk score was analyzed by multivariate Cox regression analysis to confirm whether it is a risk score or not. But beyond all that, we also investigated stratified survival analysis to detect the prognostic value of our risk score model in different subgroups. To further delve into the impact of individual target lncRNA in our prognostic risk model on KIRC patients, the relationship between expression level of each target lncRNA and clinical parameters was compared via Student's *t*-test.

### Co-expression Analysis and Immune Status Analysis

Pearson correlation coefficients were calculated to determine co-expressed lncRNA-mRNA pairs. The correlation coefficient threshold was set to >0.6, and the corresponding *P* < 0.05 was considered statistically significant. Principal component analysis (PCA) was carried out to visualize the similarities and differences among grouped samples based on the immune-related lncRNA set and whole gene expression profiles. Gene set enrichment analysis (GSEA) was implemented to determine whether an a priori defined set of genes shows statistically significant, concordant differences between the high- and low-risk groups using GSEA software version 4.0.3. C7 collection set (IMMUNOLOGIC_SIGNATURE) was downloaded from MSigDB for subsequent analysis. The stromal score, immune score, ESTIMATE score, and tumor purity were also calculated by the ESTIMATE algorithm to further explore immune cell infiltration between the low- and high-risk groups.

### Statistical Analysis

All statistical analyses were performed using R software (version 3.6.2). Differences between variables were assessed with independent *t*-tests. The association of clinicopathological variables in KIRC patients between predicted high- and low-risk cohorts was subjected to a chi-square test. The correlation was determined by Pearson or Spearman correlation analysis. Kaplan-Meier curves and log-rank tests were used to evaluate the survival data. Independent prognostic factors were assessed by univariate and multivariate Cox regression analyses. *P* < 0.05 was regarded as statistically significant.

## Results

### Filter Out Immune-Related lncRNAs Associated With Prognosis

**Figure 1:**
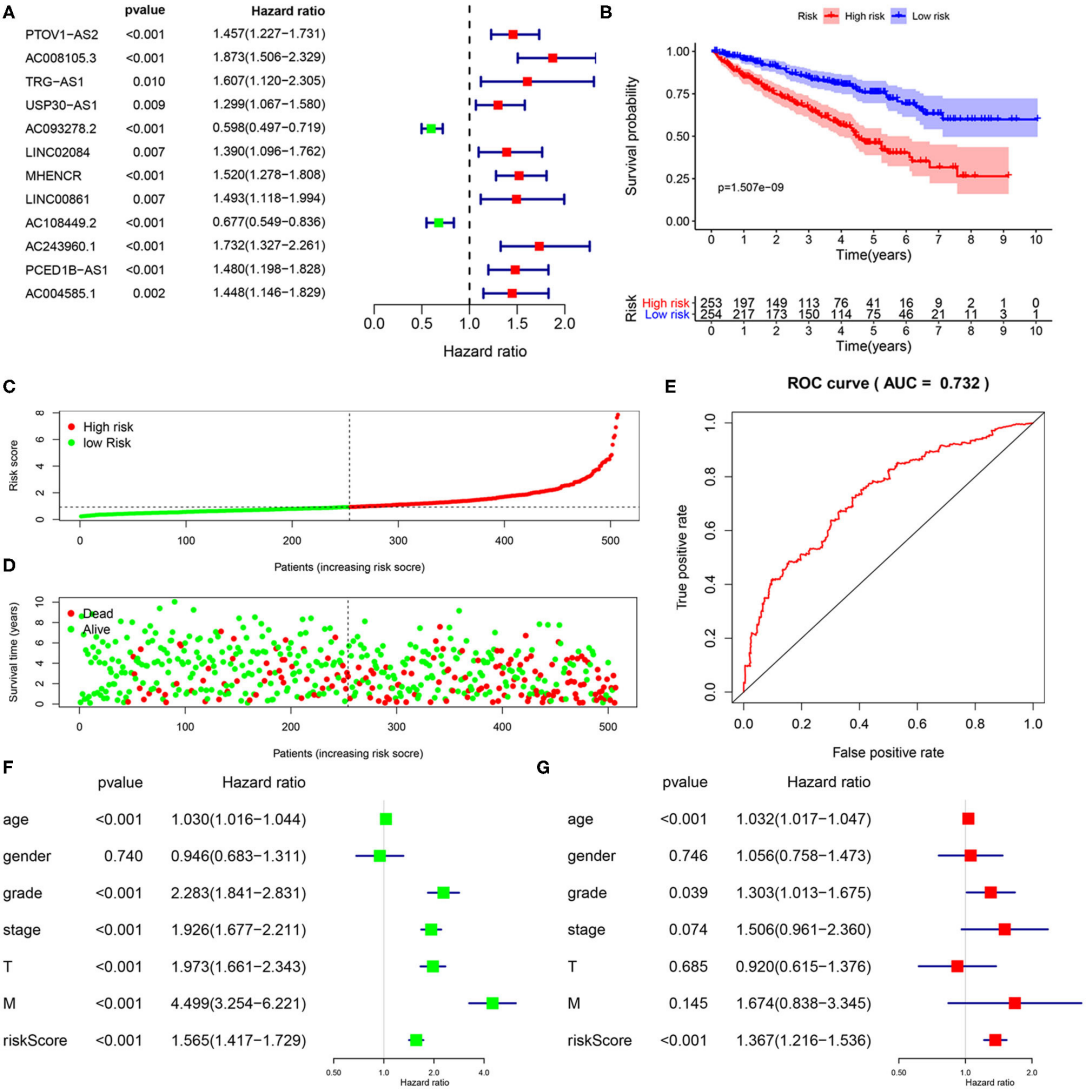
Construction and validation of immune-related lncRNA prognostic signature for KIRC. **(A)** A forest plot illustrating the HR and *P*-value from the univariate Cox regression analysis between immune-related lncRNA expression and survival data. **(B)** Kaplan-Meier overall survival curves for KIRP patients exhibited that the high-risk group suffered significantly shorter OS than those in the low-risk group. **(C)** Predictor scores of KIRP patients were sorted based on the signature. **(D)** The scatterplot of the relationship between the risk scores and the survival status/survival time. **(E)** ROC curve analysis suggests the veracity and reliability for the prognostic signature. Univariate **(F,G)** multivariate Cox regression analysis of the association between clinicopathological factors (including risk score) and overall survival of KIPC patients.

A total of 15,142 lncRNAs as well as their expression profiles were screened from the TCGA data sets, and the list of 332 immunoregulatory genes was downloaded from the Molecular Signatures Database. Then, 23 immune-related lncRNAs were screened according to the Pearson correlation analysis with the criteria of |R| > 0.8 and *P* < 0.05. Subsequently, we carried out univariate Cox regression analysis to further single out the potential prognostic lncRNAs from the cohort of immune-related lncRNAs and found that 12 lncRNAs were significantly related with the KIRC patients' OS (*P* < 0.01, Figure 1A). Remarkably, all but two of these lncRNAs (AC093278.2 and AC108449.2) were considered to be risky factors. Multivariate Cox regression analysis was then applied to confirm the optimal prognostic lncRNAs. Finally, a total of five lncRNAs were filtered out, and its regression coefficients (β) were also determined for further analysis (Table 1).

**Table 1:** The HRs, *P*-values, and Coef of 5 immune-related lncRNAs in the multivariate Cox regression analysis.

**lncRNAs**	**HR (95% CI)**	***P*-value**	**Coef**
AC008105.3	1.8743 (1.3319–2.6376)	0.0003	0.6282
AC093278.2	0.6516 (0.5222–0.8129)	0.0001	−0.4284
LINC02084	0.5019 (0.3108–0.8105)	0.0048	−0.6893
AC108449.2	0.6959 (0.5453–0.8881)	0.0036	−0.3625
AC243960.1	2.0658 (1.1331–3.7660)	0.0179	0.7255

*HR, hazard ratio; Coef, regression coefficient*.

### Construction of Five-lncRNA Prognostic Risk Signature

To further investigate whether the above five target lncRNAs could be used as prognosis biomarkers, we developed a five-lncRNA risk signature to predict the outcome of KIRC patients. Then, the risk score for each sample was calculated according to the following formula: risk score = (0.6282 × Exp_AC008105.3_) + (−0.4284 × Exp_AC093278.2_) + (−0.6893 × Exp_LINC02084_) + (−0.3625 × Exp_AC108449.2_) + (0.7255 × Exp_AC243960.1_). KIRC patients in the TCGA data sets were divided into high- (*n* = 253) and low-risk groups (*n* = 254) based on the median risk score. Significant difference was found in overall survival (OS) between the predicted two subgroups, and patients in the high-risk group suffered shorter survival time than those in the low-risk group (Figure 1B). Specifically, the 3-, 5-, and 7-year survival rates of the high-risk group were 69.2, 44.7, and 28.5%, respectively, whereas the corresponding rates in the low-risk group were 84.3, 75.1, and 62.7%. We ranked the risk scores across all KIRC patients and then analyzed their distributions according to the five lncRNAs signature-based risk scores (Figure 1C). The distributions of survival status revealed that survival rate and time of patients in the low-risk group were significantly increased compared to the high-risk group (Figure 1D). We next assessed the predictive performance of the five-lncRNA model by time-dependent receiver operating characteristic (ROC) curves. The area under the ROC (AUC) value equal to 0.732 indicated the prognostic risk model had a good predictive effect (Figure 1E). These findings imply that the prognostic risk model was competent for predicting the prognosis of KIRC patients.

### Immune-Related lncRNA Signature Was an Independent Prognostic Factor

To explore whether the five-lncRNA prognostic risk signature was independent of clinical variables, univariate and multivariate Cox regression analyses were performed with the following factors: risk score and relevant clinical factors, including age, gender, grade, AJCC stage, T stage, and M stage in the TCGA database. N stage was not analyzed for a large amount of missing data. Except the gender, all the others were significantly associated with OS in univariate analysis (Figure 1F). Results from multivariate analysis suggested risk score were still significantly linked with OS, and the five immune-related lncRNA signature could serve as an independent prognostic factor for KIRC patients (Figure 1G).

### Immune-Related lncRNA Signature Was Strongly Related With Clinical Features

**Figure 2:**
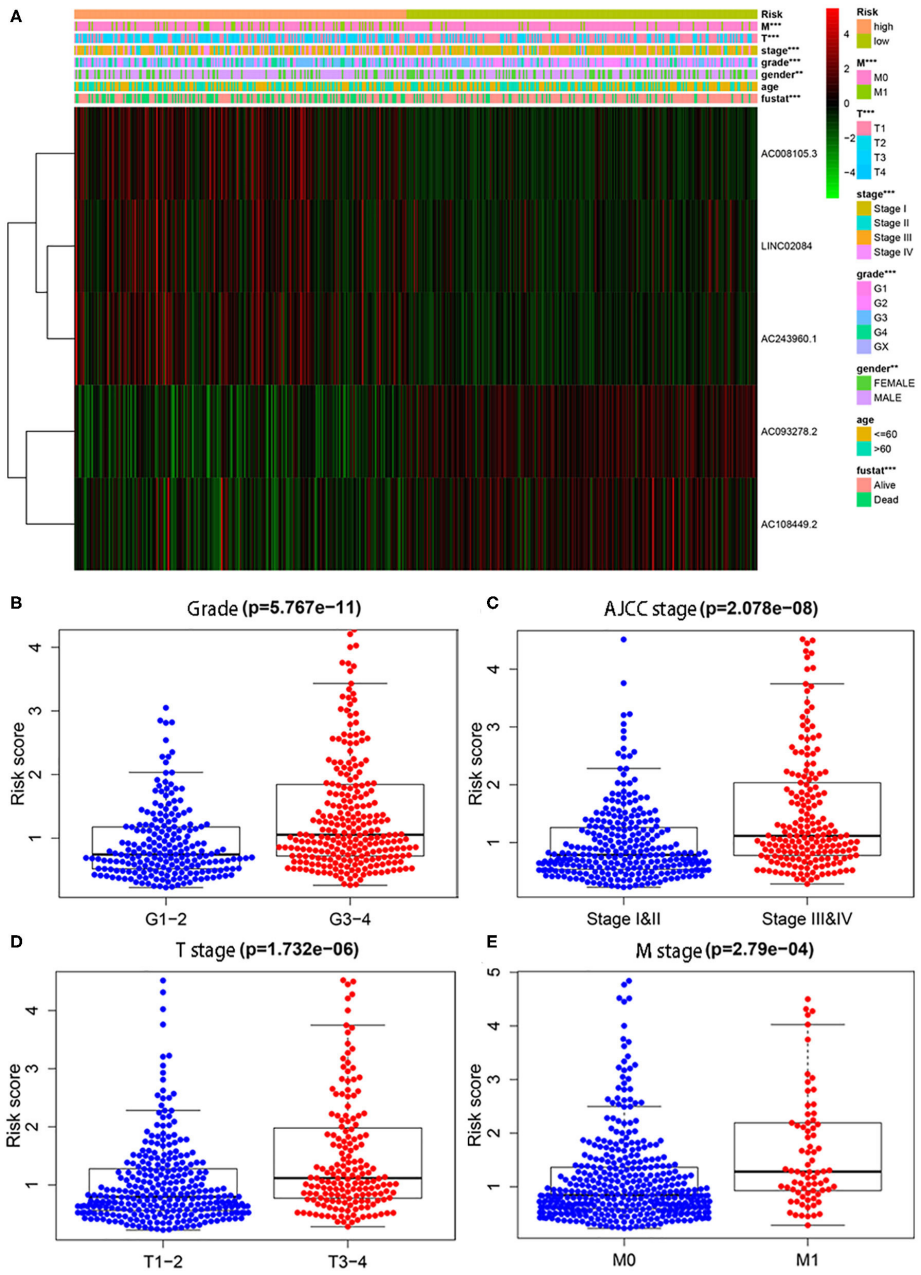
The relationship between the risk score and different clinicopathological features. **(A)** The heat map shows the distribution of clinicopathological factors and the expression of the five immune-related lncRNAs between the low- and high-risk groups. Chi-square test was used for correlation between clinical and risk. **P < 0.01 and ***P < 0.001. **(B–E)** represent grade, AJCC stage, T stage, and M stage, respectively.

In addition, we also conducted chi-square tests to investigate whether the immune-related lncRNA signature could better predict KIPC clinicopathological features. The heat map (Figure 2A) showed that there were significant differences between high- and low-risk groups in gender (*P* < 0.01), grade (*P* < 0.01), AJCC stage (*P* < 0.01), M stage (*P* < 0.01), T stage (*P* < 0.01), and survival state (*P* < 0.01). The present study further explored the relationship between the risk score and each clinicopathological characteristic, including grade (Figure 2B), AJCC stage (Figure 2C), T stage (Figure 2D), and M stage (Figure 2E). As expected, we discovered that high-grade and advanced-stage tumors were significantly associated with the high-risk group, and low-grade and early stages were related with the low-risk group (Figure 2). The results show the immune-related lncRNA signature may serve a pivotal role in oncogenesis and tumor progression of KIRC.

**Figure 3:**
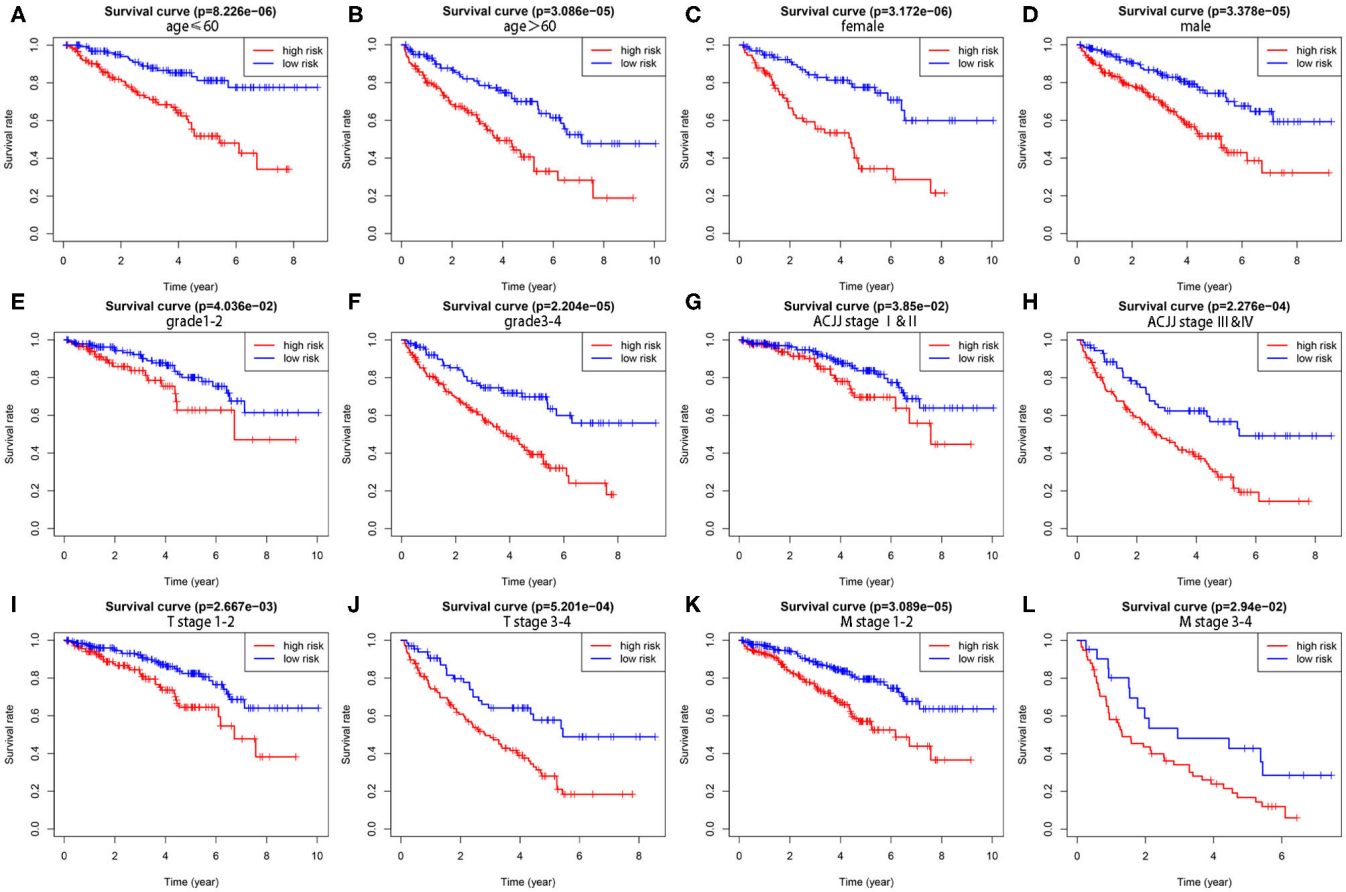
The survival differences between high- and low-risk KIRP patients stratified by clinical factors. **(A,B)** The difference in OS stratified by age (age ≤ 60, age > 60) between two groups. **(C,D)** The difference in OS stratified by gender (male, female) between two groups. **(E,F)** The difference in OS stratified by grade (G1-2, G3-4) between two groups. **(G,H)** The difference in OS stratified by AJCC stage (Stage I/II, Stage III/IV) between two groups. **(I,J)** The difference in OS stratified by T stage (T1-2, T3-4) between two groups. **(K,L)** The difference in OS stratified by M stage (M0, M1) between two groups.

To demonstrate the widespread utility of the signature, we further carried out the stratification analysis using the following clinical variables: age (<60 and ≥60), gender (female and male), tumor grade (G1-2 and G3-4), AJCC stage (I & II and III & IV), T stage (T1-2 and T3-4), and M stage (M0 and M1). Importantly, as we show in Figure 3, survival analysis indicates that the signature has predictive significance for all hierarchical cohorts. The low-risk group patients had significantly better survival compared to high-risk group patients for each subgroup. In sum, these results testify that the five-lncRNA prognostic risk signature might exert critical roles in determining the prognosis of KIRC patients.

**Figure 4:**
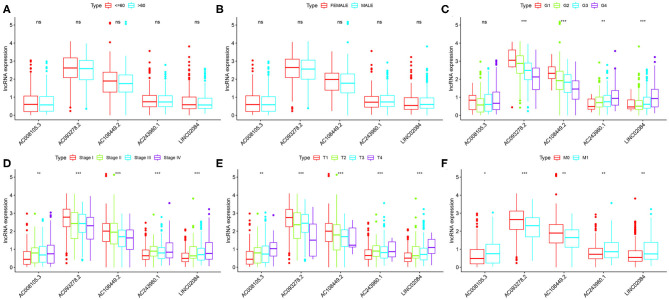
Correlation between the expression level of a single lncRNA in the signature and clinical variables. **(A–F)** represent age, grade, grade, AJCC stage, T stage, and M stage, respectively. NS, not significant. *P < 0.05, **P < 0.01, and ***P < 0.001.

Finally, we compared the correlation between the expression level of a single lncRNA in the signature and clinical variables to deeply explore the impact of target lncRNAs on KIRC. In terms of age alone, there was no significant difference in the distribution of expression levels of all five lncRNAs (Figure 4A). The same results were found for gender (Figure 4B). As for different KIRC grades, AC243960.1 and LINC02084 were increased with tumor grade, and AC093278.2 and AC108449.2 were decreased. No significantly different in the expression values of AC008105.3 was detected between different tumor grades (Figure 4C). All five immune-related lncRNAs are considered to exert their effects in AJCC stag (Figure 4D), T stage (Figure 4E), and M stage (Figure 4F) to a certain degree. In general, the expression levels of AC008105.3, AC243960.1, and LINC02084 were positively correlated with tumor staging, and AC093278.2 and AC108449.2 were negatively correlated with tumor staging, which was consistent with the above study.

### Somatic Mutations in Different Subgroups Based on Immune-Related lncRNA Signature

**Figure 5:**
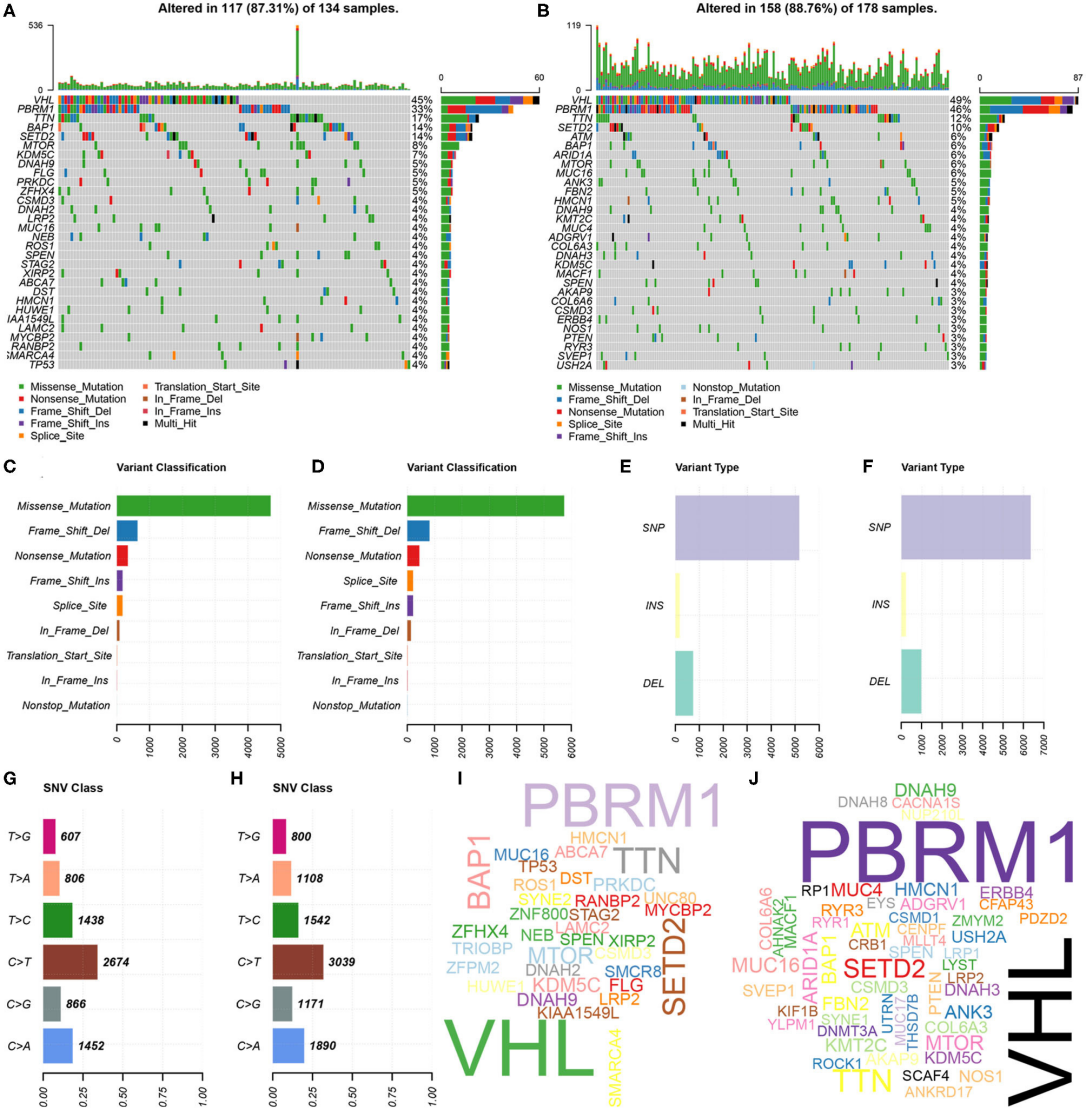
Landscape of mutation profiles between high- and low-risk KIRP patients. **(A,B)** Waterfall plots represent mutation information in each sample of the high- and the low-risk group KIRP patients. The diverse colors with annotations at the bottom represent the various mutation types. **(C,D)** The variant classification in high- and the low-risk group KIRP patients. **(E,F)** The type of genetic alterations in high- and the low-risk group KIRP patients. **(G,H)** The SNV class in high- and the low-risk group KIRP patients. **(I,J)** The gene cloud plot showed the mutated frequencies in high- and the low-risk group KIRP patients. The larger the gene, the higher the mutation frequency.

Further, the somatic mutation profiles of 336 KIRC patients were utilized to explore common somatic mutations in high- and low-risk group patients. Among these patients, 134 (39.88%) belonged to the high-risk group, 178 (52.98%) belonged to the low-risk group, and the remaining 24 (7.14%) were excluded based on the above exclusion criteria. Mutation data were analyzed and visualized using the “maftools” package. Mutation information for each gene in each sample of the high- and low-risk groups were demonstrated by waterfall plots (Figures 5A,B), and we found that the top 10 mutated genes in the high-risk group were VHL, PBRM1, TTN, BAP1, SETD2, MTOR, KDM5C, DNAH9, FLG, and PRKDC, and in the low-risk group were VHL, PBRM1, TTN, SETD2, ATM, BAP1, ARID1A, MTOR, MUC16, and ANK3. Interestingly, TP53 was one of the most common mutated genes in cancer, occurring more frequently in the high- than in the low-risk group. In addition, mutations were further sorted based on the different classifications in detail, and missense mutations are the biggest fraction among these mutations in both groups (Figures 5C,D). The most frequently mutation type in both groups was single nucleotide polymorphism (SNP) (Figures 5E,F), and C > T transversion accounted for the most common of single nucleotide variants (Figures 5G,H). Gene cloud plots showed the mutated frequencies of other genes (Figures 5I,J).

### lncRNA-mRNA Co-expression Network Analysis

**Figure 6:**
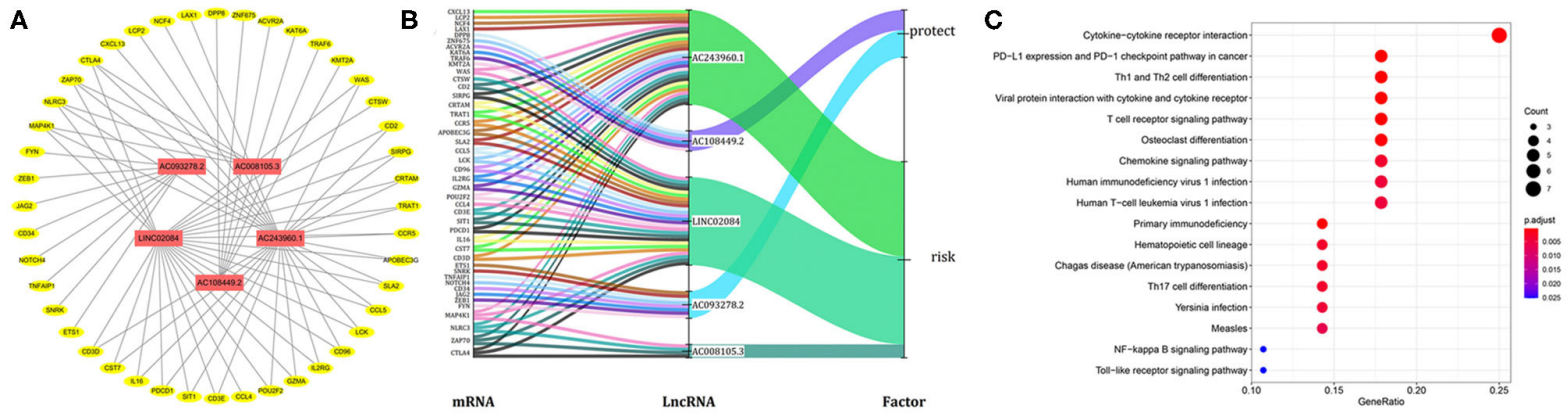
Functional annotation analysis of the five-immune-related lncRNAs prognostic signature according to co-expressed lncRNA-mRNA. **(A)** The lncRNA-mRNA co-expression regulatory network based on five immune-related lncRNAs and their highly related genes (|R| > 0.6, *P* < 0.05). **(B)** A Sankey diagram was depicted to visualize the co-occurrences of lncRNAs, mRNAs, and factors. **(C)** Results for KEGG enrichment analysis of the mRNAs co-expressed with five lncRNAs.

Considering that lncRNA and miRNA can affect the development of tumors through mutual regulation, the lncRNA-mRNA co-expression relationship network was constructed using Cytoscape software. As shown in Figure 6A, we found that these five target lncRNAs had obvious correlation with 44 mRNAs (|R| > 0.6 and *P* < 0.05). A Sankey diagram was depicted to visualize the co-occurrences of lncRNAs, mRNAs, and factors (Figure 6B). Results suggest that AC243960.1 and LINC02084 may be the major components among lncRNAs, as are CTLA4, ZAP70, NLRC3, and MAP4K1 in mRNAs. In addition, 72 significantly co-expressed lncRNA-mRNA pairs were identified as relevant. And among them all, MAP4K1, involved in regulation of the mitogen-activated protein kinase (MAPK) signaling pathway, was the closest correlation with AC243960.1. According to the KEGG analysis for mRNAs co-expressed with five lncRNAs, as expected, we observed that the majority of the enriched pathways manifested the immunomodulatory functions, and the top five significantly enriched pathways involved in cytokine–cytokine receptor interaction, PD–L1 expression, and PD−1 checkpoint pathway in cancer, Th1 and Th2 cell differentiation, viral protein interaction, with cytokine and cytokine receptor as well as T cell receptor signaling pathway (Figure 6C).

### Analysis of Immune Status Between Low- and High-Risk Groups

**Figure 7:**
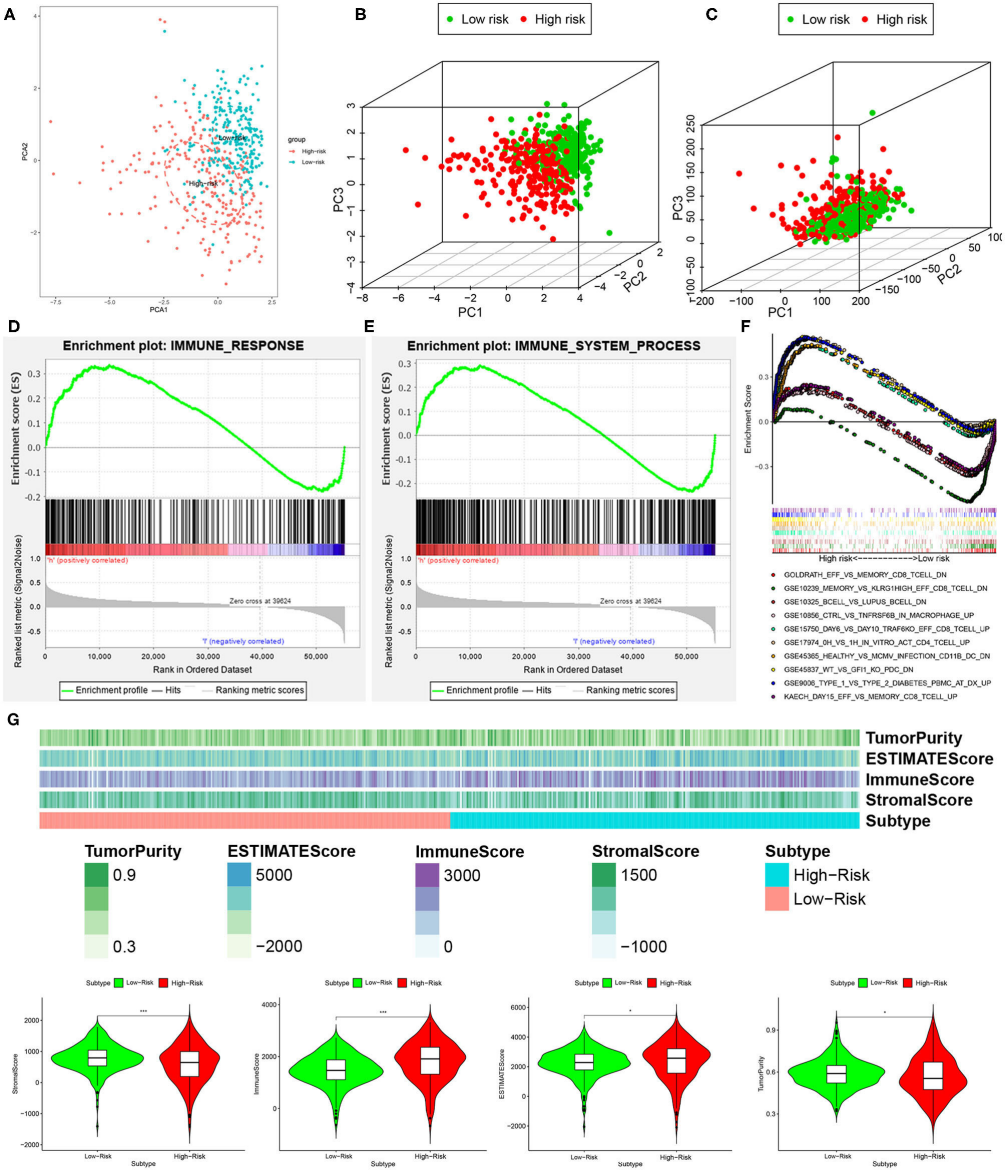
High- and low-risk groups showed different immune status. **(A,B)** Principal component analysis for immune-related lncRNAs sets between the high- and low-risk groups, showing a remarkable difference between two patterns. **(C)** Principal component analysis for genome-wide expression profiles between high- and low-risk groups, no significant separation in the immune status between two patterns. GSEA analysis exhibited that both IMMUNE_RESPONSE **(D)** and IMMUNE_SYSTEM_PROCESS **(E)** were enriched in the high- compared with low-risk group. **(F)** The five most significant gene sets in C7 collection sets (IMMUNOLOGIC_SIGNATURE) between two groups. **(G)** The high-risk group had a lower stromal score and tumor purity but higher immune score and Estimate score compared with the low-risk group. *P < 0.05 and ***P < 0.001.

We performed principal component analysis (PCA) to further assay the distinct distribution between high- and low-risk groups using the immune-related lncRNA set and whole gene expression profiles. As a result, the samples tended to be sorted into two sections, and the immune status of KIRC patients in the high-risk group was significantly different from those in the low-risk group according to immune-related lncRNAs sets (Figures 7A,B). However, there was no significant separation in the immune status of each group when PCA was done based on the genome-wide expression profiles (Figure 7C).

Furthermore, GSEA analysis was performed, and the results exhibited that both IMMUNE_RESPONSE (Figure 7D) and IMMUNE_SYSTEM_PROCESS (Figure 7E) were enriched in the high-risk group compared with the low-risk group. For the present study, we also used C7 collection sets (IMMUNOLOGIC_SIGNATURE) for GSEA analysis to further analyze differentially expressed genes. We observed that a total of 4,281 gene sets were significantly enriched (cutoff FDR < 0.25 and NOM *P* < 0.05). Among them, 709 and 3,572 gene sets were significantly enriched in the high- and low-risk groups, respectively. The five most significant gene sets in the high- and low-risk groups are shown in Figure 7F.

Besides this, to further explore immune cell infiltration between the low- and high-risk groups, we calculated stromal score, immune score, ESTIMATE score, and tumor purity according to the ESTIMATE algorithm. The high-risk group had lower stromal score and tumor purity but higher immune score and ESTIMATE score compared with the low-risk group (Figure 7G). In a word, a five-lncRNA prognostic risk signature was closely related to the immune status of KIRC patients, and the different immune status was showed between the low- and high-risk groups.

### Validation of the Expression Levels of Those Five lncRNAs Between Tumor and Normal Samples

**Figure 8:**
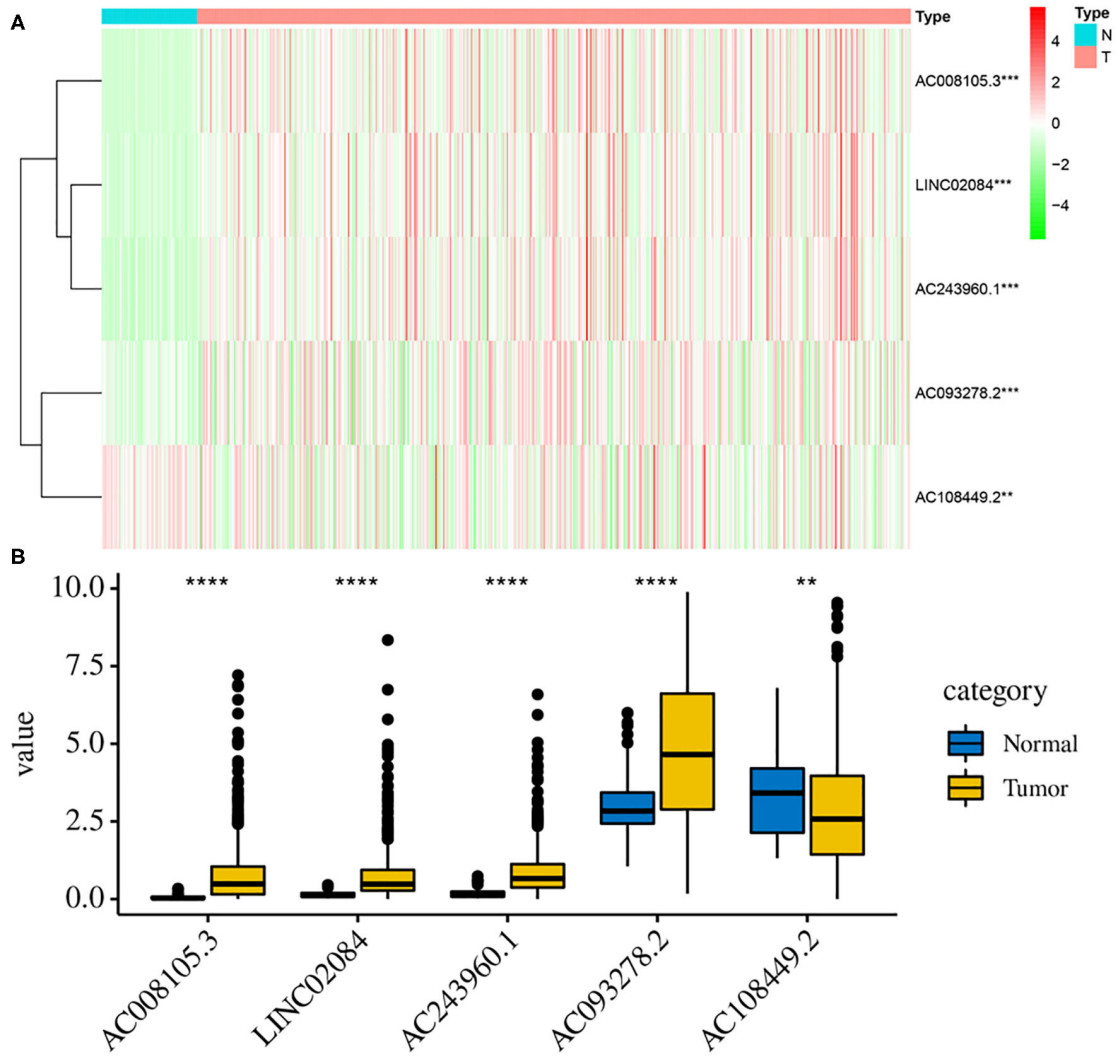
Validation the expression levels of those 5 lncRNAs between tumor and normal samples. The heat map **(A)** and bar graph **(B)** showed the expression levels of 5 lncRNAs between 539 KIRC tumor tissues and 72 non-tumor tissues in the TCGA data set. The mean expression levels of AC008105.3, LINC02084, AC243960.1, and AC093278.2 in KIRC samples were significantly lower while AC108449.2 was significantly higher than that in non-tumor tissues. **P < 0.01, ***P < 0.001, and ****P < 0.0001.

Additionally, to further verify our analysis, the expression levels of five lncRNAs were assessed in 539 KIRC tumor tissues and 72 non-tumor tissues in the TCGA data set. The mean expression levels of AC008105.3, LINC02084, and AC243960.1 in KIRC samples were significantly lower, nd AC108449.2 was significantly higher than that in non-tumor tissues (Figures 8A,B), which were consistent with our analysis findings. The results proved the reliability of our analysis. However, it was interesting that AC093278.2 was considered to be a protective factor on the above analysis but was represented significantly higher in KIRC samples than in non-tumor liver samples. This may be because AC093278.2 could exert various functions at different stages of KIRC tumorigenesis and development.

## Discussion

Although the efficacy of surgical resection had been proven to be central to the cure for localized RCC and achieved high cure rates, the treatment outcome for advanced and metastatic RCC remains unsatisfactory (24). For some KIRC patients with similar clinical risk factors, their responses to treatment and prognosis are different due to molecular heterogeneity (25). Thus, in addition to traditional clinical risk factors, identifying additional molecular prognostic indicators is imperative. Previous research has reached a consensus that the immune system plays complex and extensive roles in both the positive and negative regulation of tumor development and progression (26). Correspondingly, lncRNAs are emerging as critical regulators of gene expression in the immune system (17). It is worth noting that immune-related lncRNAs may be more highly expressed in immune cells and are significantly correlated with immune cell infiltration (14).

In the current study, 332 immunoregulatory genes were obtained from two immune-related pathways for further subsequent analysis. One of the major findings in our study was that we constructed a five immune-related lncRNA signature and verified its reliability and stability through a time-dependent ROC curve. In addition, we observed that KIRC samples with a good or poor prognosis could be distinguished based on the signature generated by these lncRNAs. Univariate and multivariate Cox regression analyses demonstrated that the signature was an independent prognostic predictor for OS in KIRC patients. We further investigated stratified survival analysis for different clinicopathological parameters to verify wide applicability of the signature and discovered that the signature could also divide KIRC samples into high-risk groups with shorter OS and low-risk groups with longer OS in different subgroups. Additionally, we compared correlation between the expression level of a single lncRNA in the signature and clinical variables and confirmed that, among these lncRNAs, AC008105.3, LINC02084, and AC243960.1 were risk-associated genes, and AC093278.2 and AC108449.2 were regarded as protective genes. PCA suggested that the five immune-related lncRNA set drew a clear distinction between high- and low-risk groups based on the immune-related lncRNAs compared with whole gene expression profiles. Furthermore, GSEA analysis was performed, and the results exhibited differentially expressed genes between the high- and low-risk groups. These findings indicate the value of the five immune-related lncRNAs signature for KIRC patients' prognosis and may be beneficial for clinicians to more precisely identify patients with high-risk scores, develop novel therapeutic strategies, and further potentially improve patient prognosis.

Undoubtedly, lncRNAs may contribute to the development of different tumors (including KIRC) via diverse mechanisms. Previous studies have reported that elevated expression of MRCCAT1, ATB, and SNHG14 in KIRC were correlated with poor prognosis, and this is also the case for low expression of OTUD6B-AS1 and ADAMTS9-AS2. More specifically, lncRNA MRCCAT1 promotes metastasis of KIRC via inhibiting NPR3 and activating p38-MAPK signaling (27). Song et al. implied high expression of lncRNA ATB could accelerate the proliferative and migratory rates of RCC cells and inhibit cell apoptosis through downregulating p53 via binding to DNMT1 (28). Another study revealed that SNHG14 is a critical lncRNA that promotes KIRC migration and invasion via sponging miR-203 and elevating N-WASP (29). On the other hand, Wang et al. demonstrated that the antioncogenic effect of OTUD6B-AS1 is partly mediated through the inhibition of the activity of the Wnt/β-catenin pathway and the EMT-related pathway (30). As reported previously, lncRNA ADAMTS9-AS2 inhibits the progression and impairs the chemoresistance of KIRC via miR-27a-3p-mediated regulation of FOXO1 (31). Despite some progress achieved in the field of lncRNA research, the functions of most lncRNAs still remain elusive, and the detailed molecular mechanism requires further investigation.

Recently, immunotherapy has gained more attention as a new paradigm in cancer treatment (32). In this article, the lncRNA-mRNA co-expression relationship network was further analyzed to dig deeper into the function of related lncRNAs, which is of great significance for innovation of immunotherapy strategies. The GSEA analysis was performed, and the results exhibited that both IMMUNE_RESPONSE and IMMUNE_SYSTEM_PROCESS were enriched in high-risk groups. Additionally, C7 collection sets (IMMUNOLOGIC_SIGNATURE) were used to further analyze differentially expressed genes and verify the effectiveness of the signature. It has been shown that immune infiltration was closely associated with the therapeutic responsiveness and prognosis of KIRC patients (33). Therefore, the five immune-related lncRNAs may serve as potential immunotherapy targets of KIRC.

Given that immunotherapy is emerging as a promising approach for cancer treatment, our studies have the advantage of comprehensive analysis of high-throughput sequencing data and construction of the immune-related lncRNA signature with predicting prognosis. These results and conclusions could provide significant clues for thorough dissection of lncRNAs in future experimental work. Nevertheless, several limitations of this pilot study should be acknowledged. The differences between normal and tumor samples that are visible to the immune system is essential for cancer immunotherapy and should be further analyzed. In addition, the construction and evaluation of the model depended on the public database, which requires additional experimental (for example, immunohistochemistry, PCR, and flow cytometry) and clinical data to verify our results. More research should also focus on the detailed relationship between the expression level of immune-related lncRNA and the immunophenotype.

In conclusion, we here systematically identified a five immune-related lncRNA signature, which may be beneficial for clinicians to more precisely identify patients with high-risk and further potentially improve prognosis of KIRC patients. In addition, the signature may serve as potential immunotherapy targets for the research of the molecular mechanisms.

## Data Availability Statement

Publicly available datasets were analyzed in this study. This data can be found here: the TCGA database (https://cancergenome.nih.gov/) and the Molecular Signatures Database v7.1 (MSigDB, https://www.gsea-msigdb.org/gsea/index.jsp).

## Ethics Statement

Ethical review and approval was not required for the study on human participants in accordance with the local legislation and institutional requirements. Written informed consent for participation was not required for this study in accordance with the national legislation and the institutional requirements.

## Author Contributions

ZS was responsible for study design, data acquisition, and analysis and was a major contributor to writing the manuscript. CJ was responsible for the bioinformatics analysis and the statistical analysis. CX was responsible for the figures and tables. TL was responsible for the integrity of the entire study and manuscript review. All authors read and approved the final manuscript.

## Conflict of Interest

The authors declare that the research was conducted in the absence of any commercial or financial relationships that could be construed as a potential conflict of interest.
